# Joint linkage and association analysis for identification of potentially causal polymorphisms in GAW15 data

**DOI:** 10.1186/1753-6561-1-s1-s36

**Published:** 2007-12-18

**Authors:** Joanna M Biernacka, Pimphen Charoen, Heather J Cordell

**Affiliations:** 1 Institute of Human Genetics, Newcastle University, International Centre for Life, Central Parkway, Newcastle upon Tyne, NE1 3BZ, UK; 2 University of Cambridge, Diabetes and Inflammation Laboratory, Department of Medical Genetics, CIMR, Addenbrookes Hospital, Cambridge, CB2 2XY, UK

## Abstract

In a small chromosomal region, a number of polymorphisms may be both linked to and associated with a disease. Potentially directly associated causal loci may be distinguished from indirectly associated loci by determining which associations can explain the observed linkage signal. We apply methods for testing whether association with a particular polymorphism or haplotype can explain an observed linkage signal to the Genetic Analysis Workshop 15 simulated (Problem 3) data, to try to identify potentially causal polymorphisms. We compare the power of several methods for testing the null hypothesis that association with a particular variant can explain the observed linkage signal, and discuss scenarios under which the various methods may be advantageous.

## Background

Genetic mapping studies often reveal a region of linkage containing a number of associated polymorphisms. A marker may be associated with the disease either because it has direct influence on disease susceptibility (i.e., it is a "causal" polymorphism), or because it is in linkage disequilibrium (LD) with a causal polymorphism. Distinguishing polymorphisms that may be directly associated with the trait from those that are indirectly associated due to LD with a causal variant is an important problem that may be addressed by trying to identify the polymorphism(s) that can explain an observed linkage result. If a particular locus is the only causal polymorphism in the region, then association with this locus should be able to explain all the linkage in the region. If the variant is not the causal variant, or is not the only causal variant in the region, evidence of linkage should exceed that explained by the association with this variant. Several methods have been proposed that may help identify polymorphisms that can explain an observed linkage signal. In particular, methods proposed by Sun et al. [1], Li et al. [2] and Biernacka and Cordell [3] test the null hypothesis that a particular variant can explain all of the observed linkage versus the alternative that it cannot. Rejection of this null hypothesis leads to the conclusion that other causal variants exist in the region.

If a particular locus is the only causal variant in the region, then conditional on the genotypes at that locus for the affected individuals, there should be no unexplained identical-by-descent (IBD) oversharing in the region among affected persons. Sun et al. [1] showed that under the null hypothesis that the candidate single-nucleotide polymorphism (SNP) is the sole causal site in the region, IBD sharing of affected sib pairs (ASPs) at the candidate SNP, given their genotypes at this SNP, is independent of their affected status and depends only on their genotypes at the SNP. Based on this property they proposed test statistics similar to several well known allele-sharing-based linkage statistics.

The method proposed by Li et al. [2] is based on joint modelling of linkage and association. Assuming one causal SNP in the region of linkage, they modelled the likelihood of the marker data conditional on the trait data for a sample of ASPs, with disease penetrances and disease-SNP haplotype frequencies as parameters, and proposed likelihood-ratio tests to characterize the LD between the candidate and disease SNPs. The program LAMP [4] implements the methods proposed by Li et al. [2], and has extended capabilities, including the use of parental genotype data and different types of pedigree structures. Assuming one high-risk allele and by testing each allele at the candidate locus individually, this program can also test whether association with a multi-allelic marker can explain the linkage.

Biernacka and Cordell [3] considered alternatives to the methods of Sun et al. [1] and Li et al. [2] that also condition on parental genotypes at the candidate locus/loci, and extended these approaches to tests of whether a haplotype composed of two tightly linked SNPs can explain all the linkage in a region. We refer to these modified methods proposed by Biernacka and Cordell [3] as Li-cpg and Sun-cpg (where "cpg" is used to denote "conditional on parental genotypes"). Biernacka and Cordell's implementation of the Sun-cpg method [3] can also be applied to multiple fully or incompletely linked loci to test whether association with a given set of polymorphisms can explain the observed linkage at a specified location.

## Methods

The Problem 3 data were analyzed without knowing the data-generating model. We looked at the "answers" after the analysis was complete, prior to writing the paper, and at that time carried out some additional analyses. Linkage and association analyses as well as a stepwise conditional logistic regression approach were used to select regions and SNPs for analysis. Charoen et al. [5] performed linkage and association analysis on data from Replicates 1–5, followed by analysis of SNPs and dense SNPs on chromosome 6 using the stepwise conditional logistic regression method described by Cordell and Clayton [6]. We used the results to select a number of candidate SNPs for the analysis described here.

We analysed chromosome 6 data from all replicates to test whether association with any of the candidate SNPs or the DRB1 locus can fully explain the observed linkage. Single SNPs and the DRB1 locus were analysed using LAMP [4] and the Li-cpg and Sun-cpg approaches [3]. Extensions of the Li-cpg and Sun-cpg methods were used to analyze haplotypes composed of two tightly linked markers, while sets of non-fully linked markers were analysed using the Sun-cpg method only. Under the data generating model, there were three disease loci (DRB1, C, and D) on chromosome 6. We analyzed single SNPs, the DRB1 locus, and combinations of SNPs strongly associated with disease status that were available in the data, but not the set of three true disease loci. Therefore, based on analysis of all replicates, we were able to estimate the power to reject a given SNP as the sole causal polymorphism in the region, and to estimate power of the tests of whether association with a particular haplotype or set of SNPs could explain the observed linkage signal. All tests were performed at a 5% type I error level.

## Results

### Candidate SNP selection

Details of results for linkage, association, and conditional logistic regression analyses are described by Charoen et al. [5]. Analysis of Replicates 1–5 revealed strong evidence of linkage on chromosome 6, with many SNPs in the linked region strongly associated with the disease status, including non-dense SNPs 152–155 and 162, and many of the dense SNPs. Application of forward and backward stepwise conditional logistic regression to non-dense SNPs in Replicates 1–5 always resulted in a model containing SNPs 153, 154, and 162. In most replicates, one or two other SNPs were needed to model the association with SNPs in this region. The additional SNPs significantly associated with the disease (after accounting for SNPs 153, 154, and 162) varied from replicate to replicate. Similar analysis of dense SNPs suggested that association with SNPs d3437 and d3439 could account for most of the association observed with the remaining dense SNPs in the DR/C locus region. In addition, Charoen et al. [5] found that association with at least one SNP in the vicinity of locus D (either d3931 or d3933) remained significant in all of Replicates 1–5, after accounting for the two SNPs in the DR/C locus region. These results were used to select the SNPs, haplotypes, and sets of SNPs shown in the first column of Table 1 for the analyses presented here.

**Table 1:** Results: Power

	Power (%)^b^		
	
SNP/marker/Haplotype/SNP set^a^	Li-cpg	Lamp	Sun-cpg
152	100	100	100
153	93	93	90
154	100	100	100
155	100	100	100
162	100	100	100
153, 162	--	--	100
153, 154	--	--	73
153, 154, 162	--	--	51
d3437–d3439	9	--	8
d3437–153	9	--	8
d3437–d3439, d3931	--	--	4
d3437–d3439, d3933	--	--	4
DRB1	99	100,100,100	32

^a^"SNP set" is a set of SNPs that need not be fully linked. The SNPs in the set are separated by commas. A "haplotype" is composed of two "fully linked" SNPs. The SNPs in a haplotype are shown separated by a dash.

^b^Power to reject the null hypothesis that association with the SNP/marker/SNP set/haplotype can explain all the linkage at a given location.

### Non-dense SNP analysis

Results of our analyses of all 100 replicates are summarized in Table 1. Although stepwise conditional logistic regression analysis had suggested that none of the SNPs are the sole causal variants in the HLA region [5], for SNPs 152–155 and 162 we used LAMP, Li-cpg, and Sun-cpg to test whether any one of these SNPs alone could fully explain the observed linkage signal. The aim of the analysis of all replicates was estimation of the power to reject each of these SNPs as the sole causal variant. This power depends on the underlying model parameters, including parameters that specify the effects of the causal loci, allele frequencies, and LD between the causal loci and the candidate SNPs, as well as on sample size. Using LAMP, Li-cpg and Sun-cpg, the power for SNPs 152, 154, 155, and 162 was 100%. The power for SNP 153 was 90% with Sun-cpg, and 93% with LAMP and Li-cpg.

Overall, therefore, all methods that we applied had high power to reject single SNPs as the sole causal polymorphisms on chromosome 6. We then considered several combinations of SNPs and tested whether association with a particular set of SNPs could explain the observed linkage. This question was addressed using our extension of the Sun-cpg method. For the set of SNPs {153,154} the Sun-cpg method had 73% power to reject these as the sole causal polymorphisms in the region. For the combination of SNPs 153 and 162 the power was 100%, while for SNPs 153, 154, and 162 the power was 51%.

### Dense SNP analysis

For the dense SNP sets, the Sun-cpg approach had no power (4% power) to reject the sets of dense SNPs {d3437, d3439, d3931} or {d3437, d3439, d3933} as being either causal or in complete LD with the sole causal variant(s) in the region. The likely reason for this low power was that the d3437–d3439 haplotype is in very high LD with the DRB1-C haplotype, while SNPs d3931 and d3933 are in high LD with the D locus. Thus, it is not surprising that the studied sets of dense SNPs were able to capture the association of disease with the DRB1, C, and D loci very well.

The haplotype extension of Sun-cpg had only 8% power to reject the haplotype composed of SNPs d3437 and d3439 as the sole cause of the observed linkage. Using the haplotype extension of Li-cpg, the power was 9%. The same results were obtained for the haplotype of dense SNP d3437 with non-dense SNP 153. Our analysis had little power to reject the haplotype composed of dense SNPs d3437 and d3439 alone as being either causal or in complete LD with the sole causal variant(s) in the region, although association with this haplotype does not in fact account for the effect at locus D. This is because most of the observed strong linkage in this region is due to association with the DRB1 and C loci. Hence association with the SNP d3437–d3439 haplotype, which is in high LD with the DRB1-C haplotype, can almost fully account for the observed linkage at the DRB1 locus. We also note that the haplotype methods that we had used were extensions of our Li-cpg and Sun-cpg methods, which generally have lower power than the Li and Sun methods, because of the additional conditioning on haplotypes. Extensions of the Sun and Li methods to haplotypes would be of interest, because they are expected to be more powerful than our haplotype methods. However, there are difficulties with extending those methods to haplotypes. For instance, for a haplotype extension of the Sun method, haplotype frequencies would need to be pre-specified, and those usually cannot be estimated accurately.

### DRB1 analysis

Using the Sun-cpg method for multi-allelic candidate markers [3], the power to reject the DRB1 locus as the sole causal site in the chromosome 6 region was 32%. Analysis with the program LAMP led to rejection of each of the DRB1 alleles as the sole causal allele in the region with 100% power. Analysis with the Li-cpg approach led to rejection of the DRB1 locus as the sole causal site with 99% power. However, as we explain in the Discussion, these results should be interpreted cautiously.

## Discussion

It is interesting to note that the stepwise conditional logistic approach applied by Charoen et al. [5] was much more effective at detecting the locus D effect than the methods applied here. Although Charoen et al. [5] did not study the power of stepwise conditional logistic regression in all 100 replicates, their results in the first five replicates indicate that this approach could detect the effects near the D locus as well as those near the DR/C loci (conditional logistic regression showed effects of SNPs near locus D after accounting for effects of SNPs near the DRB1-C haplotype, correctly suggesting the presence of further disease loci in the vicinity of locus D). We, on the other hand, found that a haplotype in strong LD with the DRB1-C haplotype could explain most of the observed linkage at this location, thus providing little power to detect effects near the D locus. The methods applied here are based on the contribution of causal-locus associations to linkage. Under the data generating model, locus D makes a relatively small contribution to the linkage signal in this region compared to the linkage signal from the DRB1-C effect. Although alleles at the D locus have a fairly large effect on RA susceptibility, the high-risk allele is rare. Using a single simulated replicate, we estimated that if locus D was the sole causal locus on chromosome 6, the LOD score peak for 1500 ASPs, using the six nearest STRPs and DRB1 locus, would only have been about 3.3. In Replicate 1, the LOD score peak for a similar analysis of 1500 ASPs with the same markers was approximately 95, presumably due to the much stronger effect of DR/C.

Application of the Sun-cpg method to multi-allelic markers or multiple linked markers is straightforward, although the method becomes less powerful as the candidate locus/loci become more informative for linkage [1]. LAMP [4] and the Li-cpg [3] approach can also be used to analyze multi-allelic markers. However, these methods make assumptions about the underlying disease model that are particularly limiting in the case of a multi-allelic candidate locus. LAMP assumes that only one of the alleles increases disease penetrance. The Li-cpg method also assumes that there are only two "allele-risk classes", but without the assumption that only one of the alleles belongs to the high risk class. The assumption made in the Li-cpg approach is that there is a single underlying causal SNP, which may be in complete LD with the candidate marker. Under complete LD, some of the candidate marker alleles always occur on haplotypes with the high risk form of the SNP, while all other candidate marker alleles always occur with the low risk form. The assumption in LAMP is equivalent to assuming that only one of the candidate marker alleles always occurs with the high risk SNP allele, while all other candidate marker alleles occur only on haplotypes with the low risk SNP allele. The Li-cpg method calculates a single test statistic for a multi-allelic candidate locus, while LAMP performs a separate test for each of the candidate marker alleles. The consequence of the assumptions made by LAMP and the Li-cpg method is that even if the candidate marker is the sole causal variant in the region, we may reject the null hypothesis as a result of failure of these assumptions. We may interpret the result of such as a test as rejecting the null hypothesis that the multi-allelic marker is in complete LD with the sole causal SNP in the region, assuming the particular model tested by the method. However, we must be cautious not to interpret the result as indicating that the candidate is not the sole causal variant in the region. To demonstrate this concept, we carried out a small simulation in which data were generated under a model with a single causal multi-allelic marker. We assumed four alleles with "allele risk factors" of 0.1, 0.15, 0.2, and 0.3, respectively. Genotype risks were obtained by multiplying the risk factors for two alleles. We generated 1000 data sets, consisting of 1000 ASPs each. In our simulation, the Sun-cpg approach had the correct 5% type I error (empirical type I error = 0.053 in 1000 replicates). LAMP analysis led to rejection of the null hypothesis for each of the four alleles in 100% of the replicates. This result should not be incorrectly interpreted as indicating that this is not the sole causal locus. With the Li-cpg approach, the null hypothesis was rejected in 4.5% of replicates, which is consistent with the nominal type I error of 5%. Thus it appears that a correct type I error was achieved despite the violated assumption that the four alleles can be categorized into two risk classes. This robustness to the failure of the assumption is due to the way significance in the Li-cpg approach is evaluated by simulation, with the parental and children's candidate locus genotypes fixed at the observed values [3].

As discussed above, the Sun-cpg method extends more easily to multi-allelic markers because it makes no assumptions about the underlying mode of inheritance. The model-based approach implemented in LAMP [4] and the Li-cpg method require assumptions to be made about the number of disease polymorphisms in the region and the number of alleles at these loci. Having made these assumptions, genotype relative risks and LD parameters can be estimated. These parameter estimates can contribute significantly to our understanding of the disease model and the possible role of a candidate locus. In our application of LAMP and the Li-cpg approach to the Genetic Analysis Workshop 15 data we focused on hypothesis testing rather than estimation. Further study of the properties of the estimators produced by these methods would be of interest.

## Conclusion

Analysis of the simulated data demonstrated that methods for joint modelling of linkage and association can have high power to reject the hypothesis that a single SNP is the sole causal variant in a region. However, the analyses also showed that for complex underlying models, power to reject association with a haplotype as being able to explain all observed linkage can be low. Existing methods have a number of limitations. For example, for multi-allelic candidate markers, the program LAMP cannot be used to test the null hypothesis that the candidate is the sole causal variant in the region, due to the strong assumptions made about the underlying genetic model. Nevertheless, approaches that combine linkage and association information have great potential to lead to a better understanding of the underlying causal variant effects.

## Competing interests

The author(s) declare that they have no competing interests.
